# Characterization of Niphatenones that Inhibit Androgen Receptor N-Terminal Domain

**DOI:** 10.1371/journal.pone.0107991

**Published:** 2014-09-30

**Authors:** Carmen A. Banuelos, Aaron Lal, Amy H. Tien, Neel Shah, Yu Chi Yang, Nasrin R. Mawji, Labros G. Meimetis, Jacob Park, Jian Kunzhong, Raymond J. Andersen, Marianne D. Sadar

**Affiliations:** 1 Genome Sciences Centre, British Columbia Cancer Agency, Vancouver, British Columbia, Canada; 2 Chemistry and Earth, Ocean & Atmospheric Sciences, University of British Columbia, Vancouver, British Columbia, Canada; Northern Institute for Cancer Research, United Kingdom

## Abstract

Androgen ablation therapy causes a temporary reduction in tumor burden in patients with advanced prostate cancer. Unfortunately the malignancy will return to form lethal castration-recurrent prostate cancer (CRPC). The androgen receptor (AR) remains transcriptionally active in CRPC in spite of castrate levels of androgens in the blood. AR transcriptional activity resides in its N-terminal domain (NTD). Possible mechanisms of continued AR transcriptional activity may include, at least in part, expression of constitutively active splice variants of AR that lack the C-terminal ligand-binding domain (LBD). Current therapies that target the AR LBD, would not be effective against these AR variants. Currently no drugs are clinically available that target the AR NTD which should be effective against these AR variants as well as full-length AR. Niphatenones were originally isolated and identified in active extracts from *Niphates digitalis* marine sponge. Here we begin to characterize the mechanism of niphatenones in blocking AR transcriptional activity. Both enantiomers had similar IC50 values of 6 µM for inhibiting the full-length AR in a functional transcriptional assay. However, (S)-niphatenone had significantly better activity against the AR NTD compared to (R)-niphatenone. Consistent with niphatenones binding to and inhibiting transactivation of AR NTD, niphatenones inhibited AR splice variant. Niphatenone did not affect the transcriptional activity of the related progesterone receptor, but slightly decreased glucocorticoid receptor (GR) activity and covalently bound to GR activation function-1 (AF-1) region. Niphatenone blocked N/C interactions of AR without altering either AR protein levels or its intracellular localization in response to androgen. Alkylation with glutathione suggests that niphatenones are not a feasible scaffold for further drug development.

## Introduction

Recurrence of prostate cancer after primary therapies occurs in approximately 20% of patients. These recurrent patients receive androgen ablation therapy that causes a temporary reduction in tumor burden, but the malignancy will eventually begin to grow again in the absence of testicular androgens to form castration-recurrent prostate cancer (CRPC). A rising titer of serum prostate-specific antigen (PSA) signifies biochemical failure and precedes clinical symptoms of the emergence of lethal CRPC. PSA is an example of a gene that is transcriptionally regulated by androgen receptor (AR). Thus there is continued transactivation of AR even though blood levels of androgen are low.

Androgens mediate their effects through the AR which is a ligand-activated transcription factor. This receptor contains several functional domains that include: the ligand-binding domain (LBD) to which androgens and antiandrogens bind; the hinge region which contains a nuclear translocation sequence; the DNA-binding domain (DBD) which binds to sequences called androgen response elements (AREs) in the enhancers and promoters of target genes; and the N-terminal domain (NTD) which contains activation function-1 (AF-1) which is responsible for most of the AR's transcriptional activity. The NTD is not a folded domain but rather intrinsically disordered or in a pre-molten globular structure [1] thereby making drug discovery to this domain extremely difficult.

In the absence of androgen, AR is complexed with chaperone proteins and located in the cytoplasm. Upon binding ligand, the receptor becomes hyperphosphoryated, translocates to the nucleus, dimerizes in an antiparallel orientation through N/C (NTD/C-terminal LBD) interactions, and interacts with other co-regulatory proteins including bridging factors and the basal transcriptional machinery on AREs of target genes to initiate transcription. AR regulates genes involved in proliferation and survival of prostate cancer cells and is a validated drug target for all stages of prostate cancer.

Current therapies directed at AR including androgen ablation (orchiectomy or LHRH agonists/antagonists, and 17-ketosteroid reductase inhibitors), and antiandrogens (bicalutamide, nilutamide, flutamide and enzalutamide) all target the AR LBD and will eventually fail [2]. Most patients succumb to metastatic CRCP within two to three years. One mechanism underlying failure to these therapies and the continued AR transactivation activity may be the expression of constitutively active splice variants of AR that lack LBD. Through screening of commercial and natural compound libraries we recently identified several small molecules that target the AR NTD. These include EPI-001 [2]–[4], sintokamides [5], and niphatenones [6].

Niphatenone B is a glycerol ether initially isolated from the marine sponge *Niphates digitalis* that represents a novel structural class of AR antagonist. Niphatenone B binds covalently to the AF-1 region of the AR NTD and blocks the proliferation of prostate cancer cells that are dependent on functional AR [6]. These characteristics make niphatenones an interesting candidate to further characterize for feasibility as a lead compound for drug development for the treatment of CRPC. Here we measure the IC50 values of each of the enantiomers in a cell-based functional assay and examine some potential mechanisms of action including: inhibition of transactivation of the AR NTD; blocking N/C interaction; interfering with ligand-binding; and altering levels or intracellular localization of AR protein. Reactivity and specificity for binding to the AR AF-1 were also investigated.

## Materials and Methods

### Reagents and antibodies

**Figure 1:**
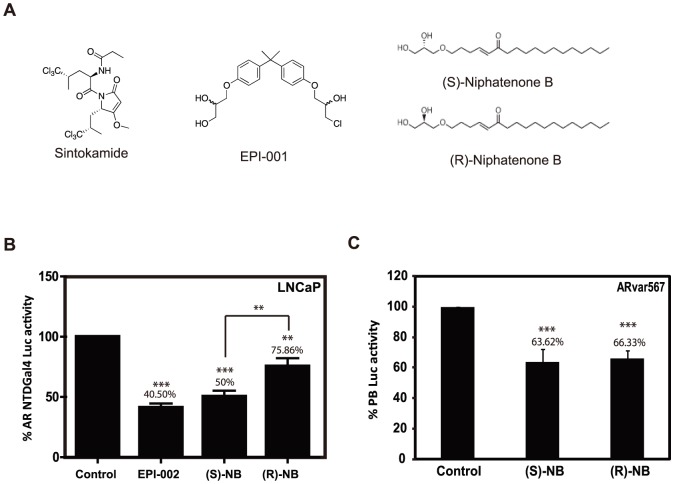
Niphatenone B enantiomers block transactivation of AR NTD. (A) Chemical structures of sintokamide, EPI-001, and niphatenone B enantiomers. (B) Transactivation assay of the AR NTD performed in LNCaP cells that were co-transfected with Gal4UAS-TATA-luciferase and AR-(1-558)-Gal4 DBD prior pre-treatment for 1 hour with 25 µM EPI-002, 7.0 µM niphatenones or DMSO vehicle control. Transactivation of AR NTD was induced by incubation with IL-6 (50 ng/ml) for 24 hours. Luciferase activity was normalized to protein concentration. (C) Niphatenones inhibit the constitutively active AR V567es splice variant. Cos-1 cells co-transfected with PB-luciferase and an expression vector for ARvar567 were treated with each niphatenone B (1 µM) or DMSO vehicle control. After 24 hours of exposure, cells were harvested and luciferase activities were normalized to protein concentrations of the samples. Data represent the mean ± SEM of n = 3 separate experiments with triplicate wells. Student's t test: **P<0.01; ***P<0.001.

(*S*)-niphatenone B or (*R*)-niphatenone B were synthesized with greater than 98% enantiometrically purity as previously reported [6] and their structures are shown on Figure. 1A. EPI-002 was made in house. The synthetic androgen, R1881 was purchased from Perkin–Elmer (Woodbridge, ON). Enzalutamide (MDV-3100) was purchased from Omega Chem, St-Romuald, Quebec. Bicalutamide was a gift from Dr Marc Zarenda, AstraZeneca Pharmaceuticals (Mississauga, ON). Interleukin-6 was purchased from R&D Systems (Minneapolis, MN). Progesterone (4-pregnene-3,20-dione), dexamethasone, HEPES, TCEP and glutathione were obtained from Sigma-Aldrich (Oakville, ON). AR PG21 antibody was purchased from Millipore (Temecula, CA). β-actin antibody was purchased from Abcam (Cambridge, MA).

LNCaP, PC3, Cos-1 and CV-1 cell lines as well as PSA(6.1 kb)-luciferase, probasin (PB)-luciferase, PRE-luciferase, GRE-Luciferase, 5xGal4UAS-TATA-luciferase and AR-(1-558)-Gal4 DBD, AR-YFP and AR^var567es^ have been described previously [3], [7].

All experiments were carried out at concentration of 7.8 µM niphatenone, 25 µM EPI-002, 1 nM R1881, and 10 µM antiandrogens (bicalutamide and enzalutamide) except where specifically indicated.

### Transfection and luciferase assay

LNCaP cells were transfected with the PB-luciferase and PSA(6.1 kb)-luciferase reporters and pre-treated the next day for 1 hour with serial dilutions of (*S*)-niphatenone B or (*R*)-niphatenone B before addition of R1881 under serum-free and phenol red-free conditions. After 48 hours of exposure, cells were harvested, luciferase activity measured and normalized to protein concentration. IC50 values were calculated using OriginPro 8.1 Software (Northampton, MA).

Transactivation of the AR NTD was measured in LNCaP cells co-transfected with the 5xGal4UAS-TATA-luciferase and AR-(1-558)-Gal4 DBD prior pre-treatment with 7.0 µM niphatenone B enantiomers or EPI-002, a positive control which binds to the AR NTD to block AR transcriptional activity [3]–[4]. Transactivation of the NTD was induced by incubation with 50 ng/ml interleukin-6 (IL6) for an additional 24 hours.

### Transcriptional activity of the AR splice variant

The expression plasmid AR^var567es^ encoding constitutively active AR splice variant that lacks the LBD was co-transfected with PB-luciferase reporter in the AR-negative Cos-1 cell line. Six hours post-transfection, cells were treated with 1 µM each niphatenone stereoisomer and incubated for another 24 hours. Luciferase activity was measured and normalized to protein concentration determined by the Bradford assay.

### Steroid receptor specificity

LNCaP cells were transfected with plasmids encoding PSA(6.1 kb)-luciferase, a progesterone receptor (PR)-beta expression vector together with PRE-luciferase or a glucocorticoid receptor (GR)-expression vector and GRE-Luciferase reporter in serum-free, phenol red-free medium. Twenty-four hours later, the cells were pre-treated with (*S*)-niphatenone B or with bicalutamide for 1 hour before addition of R1881, or progesterone (4-pregnene-3,20-dione), or dexamethasone. After 48-hours of treatment, cells were lyzed and analyzed for luciferase activities that were normalized to protein concentrations.

### Fluorescence polarization

PolarScreen Androgen Receptor, Glucocorticoid Receptor, and Progesterone Receptor Competitor Assay (Green) Kits (Life Technologies, Carlsbad, CA) were employed according to the manufacture's protocols. Serial dilution was performed for each small molecule, and solvent was compensated to ensure equal volume (v/v %) of DMSO and ethanol in each sample. Assays were done in technical triplicates using Greiner 384 black clear-bottom plates. Fluorescence polarization was measured by Infinite M1000 (TECAN, Männedorf, Switzerland) with excitation wavelength of 470 nm and emission wavelength of 535 nm.

### N to C interaction

The mammalian two-hybrid N/C interaction assay was performed using CV1 cells that were transfected with GAL4DBD-AR LBD-(AR628-919) encoding the wild type AR LBD C-terminus amino acid residues 628-919 fused to the Gal4 DBD, VP16-AR-(1-565) that encodes the VP16 transactivation domain fused to amino residues 1–565 of AR NTD and Gal4-luciferase reporter. Transfected cells were pre-treated for 1 hour with bicalutamide or (*S*)-niphatenone B before incubation with R1881 for additional 24 hours. Luciferase activity was measured and normalized to protein.

### Endogenous gene expression analysis

LNCaP cells were serum starved for 48 hours. Cells were treated for 1 hour with vehicle, (*S*)-niphatenone B or bicalutamide prior to addition of R1881 for 16 hours under serum- and phenol red-free conditions. Total RNA was isolated using RNeasy mini kit (Qiagen) and transcripts for *KLK2* and *KLK3/PSA* measured by quantitative real-time (qRT)-PCR in triplicates for each biological sample. Expression was normalized to glyceraldehyde-3-phosphate dehydrogenase (GAPDH) housekeeping gene. Primers employed were previously described [3], [8]–[9].

### Western blot analyses

LNCaP cells were serum starved for 48 hours then treated with vehicle, (*S*)-niphatenone B or bicalutamide for 1 hour prior to R1881 addition for 16 hours. Cells were harvested and whole-cell lysates were subjected to 10% SDS-PAGE. Membranes were probed with anti-AR PG21 antibody and normalized to protein levels of β-Actin.

### Nuclear translocation

LNCaP cells seeded on sterile cover slips were transfected with the expression vector for AR-YFP fusion protein. Following 24 hours incubation, cells were treated with vehicle, (*S*)-niphatenone B, bicalutamide or enzalutamide (MDV-3100) for 1 hour prior to R1881 exposure for 2 hours. Cells were fixed with paraformaldehyde, stained with DAPI, and examined by fluorescence microscopy (Zeiss, Toronto, ON).

### Click chemistry and covalent binding

Binding reactions between niphatenone B, EPI probe or DMSO vehicle control and the recombinant peptides AR AF-1 (amino acid residues 142–485) or GR AF-1 (amino acid residues 77–262) were carried out by mixing 10 µM AR AF-1 or GR AF-1 with 20 µM niphatenone B 44 or EPI-054 probes containing an alkyne group or with DMSO in equivalent amounts. Binding reaction was incubated on ice for 45 minutes. Niphatenone B or EPI probe were then labeled with fluorescein azide by Click chemistry at room temperature for 30 minutes. Proteins were separated from free probes on reducing SDS-PAGE and fluorescein was detected by an image analyzer. Coomassie blue staining was done on the same gels to show equal loading of AF-1 protein to each lane.

### Alkylation reaction

HEPES, TCEP, reference material pentachlorobenzene, and niphatenone B enantiomers (0.014 mmol) or EPI-002 (0.014 mmol), were added separately to a small vial. After deuterated DMSO was added, the sample was run NMR (0 h). Glutathione and D_2_O were added and the mixture was warmed to 37°C. The mixture was monitored by NMR every few hours (1, 2, 4, 8, 24, 72 and 99 h).

### Statistical Analysis

Values are presented as mean ± SD or ± SEM. Comparison of two populations was made by use of Student's *t* test. Analysis was performed using GraphPad Prism (version 6.01 GraphPad Sofware, Inc. La Jolla, CA).

## Results

Inhibitors of the AR NTD would be beneficial for blocking all AR species because the NTD contains the AF-1 region that is essential for AR transcriptional activity [10]. Such AR NTD inhibitors would be of clinical value for the treatments of diseases of the androgen axis such as prostate cancer. We have discovered several AR NTD inhibitors that include: decoys [11]; EPI-001 [3]–[4]; sintokamide [5], and niphatenones [6]. Synthesis and structure activity relationship studies have been completed with niphatenones A and B [6]. These compounds covalently bind to the AR AF-1 region to inhibit androgen-induced proliferation of LNCaP human prostate cancer cells at concentrations that do not block the proliferation of PC3 cells [6] that do not express a functional AR. Here we characterize the molecular mechanism of niphatenone B inhibition of AR activity and test for enantiomer specificity. The chemical structures of the two synthetic naphatenone B enantiomers have a unique pharmacophore that does not resemble other AR NTD antagonists, sintokamides or EPI-001 (Figure 1A).

### Comparable IC50 values for both enantiomers

To determine IC50 values for inhibition of AR activity by each niphatenone enantiomer, the AR-driven PB-luciferase reporter was employed that contains the androgen responsive region with two AREs. Both enantiomers had IC50 values in the range of 6 µM using this AR-driven reporter. (*S*)-Niphatenone B IC50 was 5.7±0.6 µM and (*R*)-niphatenone B IC50 was 6.3±0.5 µM. To determine whether the DNA recognition sequence (ARE and/or promoter/enhancer sequence) influenced enantiomer IC50 values, a second AR-driven reporter was tested. IC50 values using the PSA(6.1 kb)-luciferase reporter were 5.17±0.59 µM and 6.25±0.82 µM for (*S*)-Niphatenone B and (*R*)-niphatenone B respectively. Thus niphatenone B enantiomers block androgen induction of AR-driven reporters thereby supporting that they may be a new class of AR antagonist.

### Niphatenone B inhibits transactivation of the AR NTD

Niphatenone B enantiomers are effective inhibitors of full-length AR. The majority of transcriptional activity of AR resides on AR AF1 on the NTD [10], [12]–[13]. To test if niphatenone enantiomers could block AF-1 transactivation, LNCaP cells were co-transfected with Gal4UAS-TATA-luciferase and AR-(1-558)-Gal4 DBD which encodes the full NTD containing AF-1. Transactivation of the AR NTD was statistically significantly reduced by both niphatenone B enantiomers although the (*S*) configuration was significantly better than the (*R*) configuration (Figure 1B). These data suggest that niphatenones are antagonists of the AR NTD and could potentially be used to block the activities of constitutively active AR splice variants that lack LBD. To test this, Cos-1 cells that do not express endogenous AR were transiently co-transfected to ectopically express the constitutively active splice variant AR-V567es [7] along with the PB-luciferase reporter. Consistent with niphatenones blocking transactivation of the NTD, both enantiomers also inhibited the transcriptional activity of AR-v567es by approximately 40% (Figure 1C). There was no effect on the proliferation of Cos-1 cells at this concentration of niphatenones (Figure S1).

### Niphatenones B inhibit AR activity

**Figure 2:**
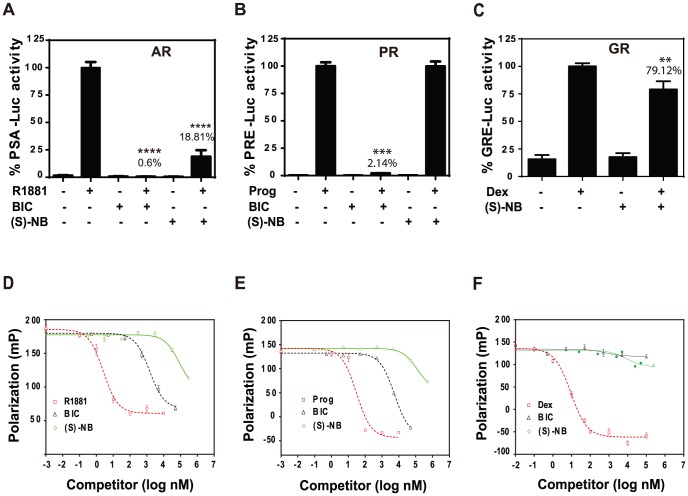
Niphatenone B inhibits AR activity. Effect of (*S*)-niphatenone B, or bicalutamide (BIC) on R1881 induced endogenous AR transcriptional activity (**A**), 4-pregnene-3,20-dione (Prog, 10 nM) induced PR transcriptional activity (B), or dexamethasone (Dex, 10 nM) induced GR transcriptional activity (C) in LNCaP cells that were transiently transfected with PSA(6.1 kb)-luciferase reporter, PRE-luciferase or GRE-Luciferase reporters and expression vector for PR-β or GR. Luciferase activities were represented as percentage of vehicle activity. Data is presented as the mean ± SEM (n = 3). Representative competition binding curve showing displacement of 1 nM fluorescently labeled ligand from recombinant AR (D), PR (E), or GR (F) LBDs (25 nM) by bicalutamide (BIC), agonist R1881, progesterone or dexamethasone, and (*S*)-niphatenone B.

Since the (*S*) configuration showed significantly better inhibition of transactivation of the AR NTD (Figure 1B), we focused on this enantiomer. To test the specificity of (*S*)-Niphatenone B for activity against the AR, the ability of (*S*)-niphatenone B to inhibit the steroidal transcriptional activity of full-length AR, and structurally related PR-β and GR were compared in LNCaP cells. As expected, the clinically used antiandrogen, bicalutamide (which binds the LBDs of both AR and PR) completely blocked androgen-induced AR (Figure 2A) and progesterone-induced PR-transcriptional activities (Figure 2B). (*S*)-niphatenone B inhibited androgen-induced AR-driven PSA-luciferase activity by approximately 80% (p<0.0001), but did not inhibit PR transcriptional activity at this same concentration. (*S*)-niphatenone B had a modest but statistically significant effect on GR-driven transcriptional activity (Figure 2C). No transcriptional activities were measured with addition of progesterone or dexamethasone in LNCaP cells not transfected with the expression vectors for PR-β or GR (Figure S2). Thus these reporters were specific to PRβ and GR for activities.

To test whether (*S*)-niphatenone B interferes with ligand-binding, we evaluated its ability to compete for the LBD of AR and related steroid receptors, PR-β and GR. At concentrations used to block the AR activity, (*S*)-niphatenone B did not interfere with ligand-binding to the AR, PR and GR LBDs as shown in fluorescence polarization assays (Figure 2D–F). However, at concentrations of 30 µM, (*S*)-niphatenone B interfered with ligand-binding. Such a result may indicate off-target effects at high concentrations.

### Niphatenones B block N/C interaction

**Figure 3:**
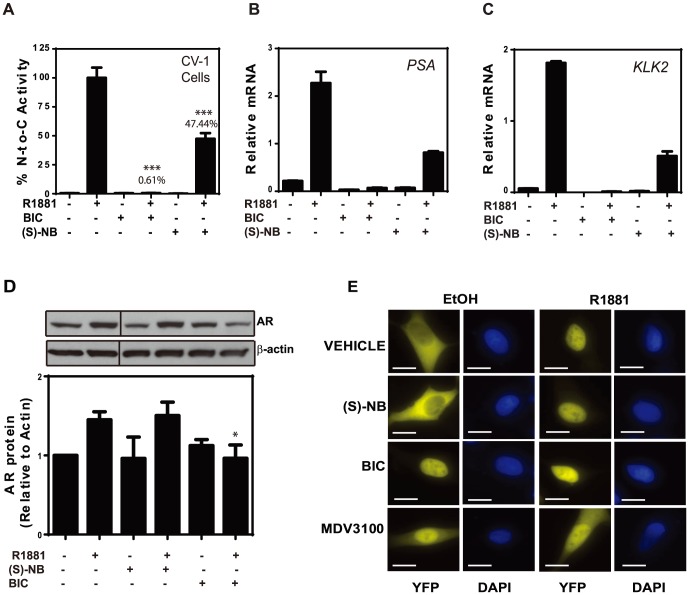
Niphatenones block N/C interaction and inhibit expression of AR regulated genes. (A) Mammalian two-hydrid assay using CV1 cells transfected with GAL4-AR DBD and/or VP16-AR TAD and Gal4-luciferase reporter. Cells were pretreated for 1 hour with bicalutamide or (*S*)-niphatenone B before addition of R1881 for 24 hours. Data represent the mean ± SEM of n = 3 separate experiments with triplicate wells. Student's *t* test: ***P<0.001. (B) *PSA/KLK3* or (C) *KLK2* mRNAs were inhibited by (*S*)-niphatenone B. LNCaP cells were pre-treated for 1 hour with (*S*)-niphatenone B, bicalutamide or vehicle prior to 16 hours incubation with R1881. Data represent the mean normalized expression (MNE) of *PSA* or *KLK2* transcripts normalized to levels of GAPDH transcripts. Representative data of n = 3 separate experiments. *P<0.05, **P<0.01, *** *P* <0.001 (Student's *t*-test). (D) (*S*)-niphatenone B does not decrease endogenous AR levels in LNCaP cells. Cells were pre-treated for 1 hour with (*S*)-niphatenone B, bicalutamide or vehicle prior to the addition of R1881 for an additional 16 hours incubation before harvesting and Western blot analysis for AR and β-actin protein levels. Samples were run on the same gel but were not contiguous (black Lines) Error bars represent the mean ± SD of n = 3 separate experiments. Student's *t* test: ***p<0.001. (E) Nuclear translocation of YFP-AR in LNCaP cells transfected with an expression plasmid encoding an AR-YFP fusion protein in serum-free conditions for 24 hours prior to pre-treatment with (*S*)-niphatenone, bicalutamide, vehicle (DMSO), MDV3100, or R1881 for 2 hours. DAPI staining indicates the location of the nucleus. Scale bar, 20 µm.

EPI-001 binds the AF-1 region in the AR NTD which blocks AR N/C interaction [3]–[4] that is required for ligand dependent activity of AR [14]–[15]. To determine if (*S*)-niphatenone B works by a similar mechanism to EPI-001, the mammalian two-hybrid system was used to measure N/C interaction in CV-1 cells that do not express endogenous AR. In the absence of androgen, no N/C interaction was detected and as expected, in its presence, androgen induced robust interaction (Figure 3A). Bicalutamide was included as a positive control and completely blocked N/C interaction. N/C interaction in response to androgen was also significantly reduced by (*S*)-niphatenone B (∼50%, p<0.0001).

### Niphatenones block endogenous expression of AR-regulated genes

Androgen induction of expression of *PSA* and *KLK2* genes are dependent on functional AR. These genes contain bone fide AREs to which the AR binds to initiate transcription. To test whether niphatenone B has an effect on endogenous gene expression, the levels of *PSA* and *KLK2* transcripts were measured in LNCaP cells in response to androgen with and without (*S*)-niphatenone B treatment. Androgen induced approximately a 9-fold increase in PSA mRNA levels but this induction was inhibited by approximately 70% by (*S*)-niphatenone B (Figure 3B). This is comparable to the 80% inhibition of PSA mRNA by 10 µM enzalutamide in LNCaP cells treated with androgen whereas 1 µM enzalutamide only caused a 30% decrease in mRNA [16]. Similarly (*S*)-niphatenone B also blocked androgen-induction of *KLK2* gene expression (Figure 3C). Together these data support that niphatenones inhibit AR transcriptional activity.

### Niphatenone B does not alter levels or intracellular localization of AR protein

The antagonist effect of niphatenones on AR activity may be mediated by alterations of levels or subcellular localization of AR protein. To begin to further reveal the molecular mechanism of action of niphatenones, LNCaP cells were exposed to (*S*)-niphatenone B in the presence or absence of androgen. In the absence of androgen, there was no significant change of the levels of AR protein in LNCaP cells with (*S*)-niphatenone B (Figure 3D, lane 3). In the presence of androgen (lanes 2, 4, and 6), levels of AR protein were also comparable between control- and niphatenone B-treated cells. Therefore the inhibitory effects of niphatenone B in the presence of androgen on AR transcriptional activity (Figure 2A, and 3B and C) are unlikely to be caused be decreased levels of AR protein. However, in the presence of androgen, cells treated with bicalutamide (lane 6) had significantly (*p* = 0.03) decreased levels of AR protein compared to androgen only (lane 2).

To determine if niphatenones altered AR subcellular localization, a plasmid encoding YFP-AR was transfected into LNCaP cells and fluorescence microscopy was used to detect the localization of this YFP-AR chimera in both the absence and presence of androgen. In the absence of androgen, niphatenone B had no effect on AR localization with the AR predominantly in the cytoplasm comparable to the vehicle-treated cells (Figure 3E). In the absence of androgen, both antiandrogens, bicalutamide and enzalutamide (MDV3100), caused the AR to become predominantly nuclear as previously reported [4], [16]–[18]. In the presence of R1881, the AR was predominantly nuclear with all treatments including niphatenone B and antiandrogens (Figure 3E).

### Niphatenone B covalently binds both AR AF-1 and GR AF-1

**Figure 4:**
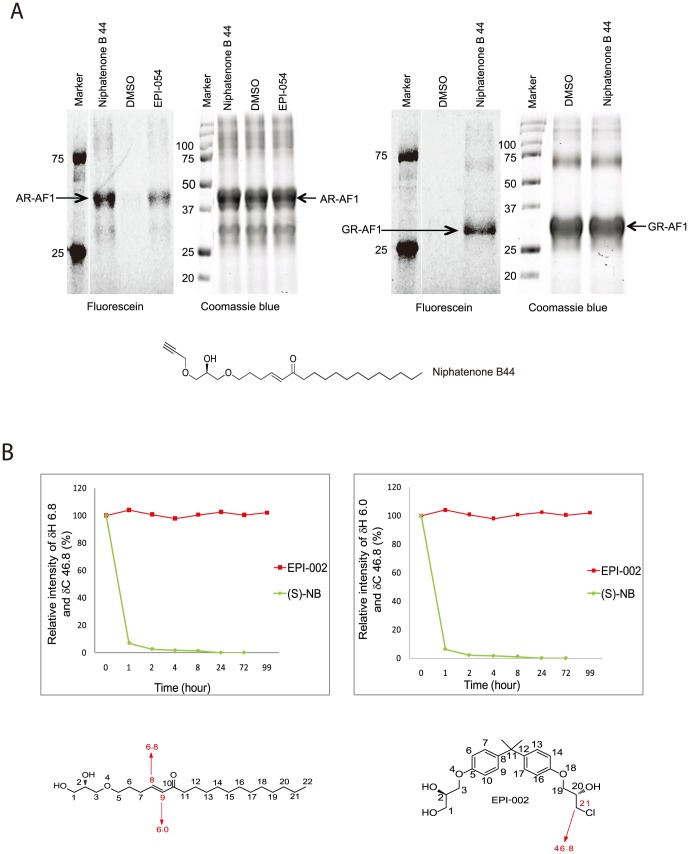
Niphatenone B covalently binds both AR AF-1 and GR AF-1. (A) AR AF-1 and GR AF-1 peptides were incubated with DMSO (vehicle), niphatenone B 44 (structure shown at bottom of the) or EPI-054 for 45 minutes prior to Click-chemistry to add a fluorescein moiety to the probe. Fluorescein-labeled probes covalently bound to the AF-1 peptides were detected with reducing SDS-PAGE. Total protein in each of the lanes was shown by Coomassie blue staining of the gel. (B) Niphatenone alkylates glutathione. A mixture of glutathione (0.07 mmol), (*S*)-niphatenone (0.014 mmol), EPI-002 (0.014 mmol) was monitored by NMR after 1, 2, 4, 8, 24, 72 and 99 hours. Proton 8 and 9 integral values were used as a reference for niphatenone and carbon 21 integral value was used a reference for EPI-002. See Tables 1 and 2.

Previously, Click chemistryusing a propargyl ether of (*S*)-niphatenone B revealed covalent binding to the AR AF-1 peptide in a cell-free assay [6]. To determine if the niphatenones might bind to other peptides, the GR AF-1 peptide (amino acids 77–262) was examined, as GR is the only steroid receptor with some limited sequence identity (77–88 residues) in the highly conserved motif within the AF-1 of AR (amino acid 236–247) [19]–[21]. An EPI-001 analogue (EPI-054) has been shown to bind covalently to the AR-AF1 region [4] and was included here as a positive control. Niphatenone Click chemistry probe (20 µM) resulted in covalent binding to both AR AF-1 and GR AF-1 peptide (Figure 4A). Equal loading of either AR AF-1 or GR AF-1 peptides to each of the lanes was shown by staining the gel with Coomassie blue.

These data together with those shown in Figure 2D–F suggest that at higher concentrations, niphatenone B may have non-specific activity. Examination of the chemical structure of niphatenone reveals an enone moiety that could be reactive and thereby cause niphatenone to be an alkylating agent. To test this, alkylation reactions using glutathione were incubated with both niphatenone B enantiomers. EPI-002 specifically and covalently binds to the AR AF-1 region and is not a random alkylator [4] and was included as a negative control. Both niphatenone B enantiomers formed adducts with glutathione (Table 1 and 2, Figure 4B). Consistent with previous reports, EPI-002 did not form adducts with glutathione.

**Table 1:** Integral Value of (*S*)-, (*R*)-Niphatenone B, and EPI-002.

Time (h)	δH6.8 (ppm)		δH6.0 (ppm)		δC46.8 (ppm)
	(*S*)-NB	(*R*)-NB	(*S*)-NB	(*R*)-NB	EPI-002
0	5.1	5.1	5.3	5.3	5.1
1	0.3	0.3	0.4	0.3	5.3
2	0.1	0.1	0.1	0.1	5.2
4	0.1	0.1	0.1	0.1	5.0
8	0.1	0.0	0.1	0.1	5.2
24	0.0	0.0	0.0	0.0	5.3
72	0.0	0.0	0.0	0.0	5.2
99	-	-	-	-	5.2

Note: Integral values were based on the reference material pentachlorobenzene.

**Table 2:** Relative Intensity of (*S*)-, (*R*)-Niphatenone B, and EPI-002.

Time (h)	δH6.8 (ppm)		δH6.0 (ppm)		δC46.8 (ppm)
	(*S*)-NB	(*R*)-NB	(*S*)-NB	(*R*)-NB	EPI-002
0	100	100	100	100	100.0
1	6.4	5.3	7.0	5.5	104.1
2	2.1	2.2	2.6	2.1	100.8
4	1.6	1.4	1.7	1.5	97.9
8	1.2	0.4	1.3	1.1	100.6
24	0.0	0.0	0.0	0.0	102.5
72	0.0	0.0	0.0	0.0	100.4
99	-	-	-	-	102.1

## Discussion

Finding novel compounds that have unique molecular mechanisms of action to block the AR is of high interest for the treatment of advanced prostate cancer. Enzalutamide is a second generation antiandrogen and abiraterone is an irreversible CYP17 inhibitor which blocks synthesis of androgens. Both of these drugs directly or indirectly target the AR LBD and have been approved by the FDA for the treatment of metastatic CRPC. Unfortunately, these new drugs only increase survival by approximately 4–5 months. A mechanism of resistance to these drugs is thought to involve the generation of constitutively active AR splice variants that lack the LBD [22]–[23]. A functional AF-1 in the NTD is essential for all AR species including both full-length AR and truncated constitutively active AR variants. Therefore a drug that inhibits the AR NTD would have potential therapeutic value for the treatment of advanced prostate cancer especially those cases that are resistant to current therapies and express constitutively active AR splice variants.

Several antagonists of the AR NTD have been discovered [3]–[6]. Niphatenones A and B were isolated from an extract of the marine sponge *Niphates digitalis* that showed activity in a cell-based assay designed to detect inhibitors of the AR [6]. The natural compounds as well as synthetic analogues were evaluated and revealed that niphatenone B covalently bound to the AF-1 region in the AR NTD, had good activity against full-length AR activated by androgen, and blocked androgen-dependent proliferation while having no effect on cells that do not express a functional AR [6]. Hence niphatenone B showed promising initial characteristics as an inhibitor of the AR NTD. However, to assess the feasibility of niphatenone B for the treatment of advanced prostate cancer and prior to in vivo efficacy studies, more work was required to provide an indication of activity against constitutively active AR splice variants, specificity, and selection of the best enantiomer.

Here niphatenone B enantiomers were characterized and revealed: 1) comparable IC50 values of approximately 6 µM for blocking full-length AR activity; 2) both enantiomers inhibited constitutively active AR splice variant v567es; 3) (*S*)-niphatenone inhibited transactivation of the AR NTD better than (*R*)-niphatenone; 4) (*S*)-niphatenone had no effect on PR activity; 5) niphatenone B did not affect the levels or intracellular localization of AR protein; 6) at higher concentrations both enantiomers had off-target effects that included blocking androgen binding to the AR LBD, forming adducts with glutathione, and covalent binding to recombinant GR AF-1 peptide.

In terms of IC50s of antiandrogens versus NTD inhibitors, these are different targets and different classes of proteins. Antiandrogens are competitive inhibitors of androgen that bind the ligand-binding pocket which is a structured folded domain maintained in an accessible conformation by chaperone proteins. AR NTD is intrinsically disordered without enzymatic activity or rigid binding pockets for receptor-ligand interaction, thus small-molecule inhibitors work by blocking of essential protein-protein interactions required for active transcriptional complexes. In vitro cell-based studies generally employ 10 µM for all antiandrogens including enzalutamide and ARN-509 to block AR activity in functional assays [16], [18]. Concentrations of 10 µM enzalutamide are used for experiments such as proliferation, ChIP, reporter assays, and mRNA transcript levels in LNCaP cells [16], [18]. Enzalutamide causes 80% inhibition of *PSA* mRNA and 86% inhibition of ARE-luciferase activity in LNCaP cells at 10 µM with only 30% inhibition of *PSA* transcripts at 1 µM [16]. AR NTD inhibitors (niphatenones, EPI-001, and sintokamides) inhibit AR-driven reporter gene constructs, *PSA* transcripts, and other functional assays at similar concentrations to enzalutamide also in LNCaP cells [3]–[6]. Thus the effective concentrations used for niphatenones and other NTD inhibitors employing these functional assays are in the same range as enzalutamide and other antiandrogens.

Niphatenone B binds covalently to the AF-1 region and inhibited transactivation of the AR NTD. More than 160 proteins have been reported to interact with the AR [24] with the NTD of this receptor considered as a hub for protein binding due to its pre-molten globular structure. Most likely niphatenone B prevents interaction of the AF-1 region with essential proteins that are required for transcriptional activity. One protein-protein interaction investigated here was N/C interaction of the AR in response to androgen that is important for retention of androgen in the ligand-binding pocket [14], [25]–[27]. The ability for niphatenone B to attenuate N/C interaction would potentially reduce the dissociation rate of androgen thereby leading to a less active full-length receptor. However, this potential mechanism cannot be considered to be the main mechanism of action for niphatenone to attenuate AR transcriptional activity since niphatenones also blocked AR splice variant activity. Splice variants lack LBD so cannot bind ligand or form N/C interactions. The ability of a small molecule inhibitor to prevent nuclear translocation of the AR or to specifically degrade AR protein would be desirable qualities for a drug candidate for the treatment of prostate cancer. Here niphatenone did not inhibit AR transcriptional activities by either of these mechanisms.

Consistent with the requirement for a functional AF-1 for a transcriptionally active receptor, niphatenones blocked the activity of both full-length AR endogenously expressed in LNCaP cells as well as the constitutively active AR splice variant, v567es. In clinical samples and laboratory models, this AR splice variant is expressed in response to castration, abiraterone and antiandrogens (including enzalutamide) and is correlated to poor survival for patients with CRPC [7], [22]–[23], [28]. Hence an antagonist of the NTD would be beneficial for prostate cancer patients that are failing current hormone therapies due to expression of constitutively active AR splice variants (Figure S3).

(*S*)-niphatenone B had no effect of PR transcriptional activity while had a modest effect (∼20%) on GR and substantially attenuated AR (>80%) transcriptional activity. However, niphatenones at concentrations above 10 µM lacked desirable specificity and have off-target binding. The first indication of potential off-targets came from the ligand-binding assay. At a concentration of 30 µM, niphatenone reduced polarization that is caused by decreased binding of fluorescently labeled hormone (fluoromone) to the LBDs of AR, PR and GR. This result suggests binding of niphatenone B to the AR-, PR- and GR-LBDs in addition to its covalent binding to AR NTD. The NTD and LBD of AR lack sequence similarities. The Click chemistry probe for niphatenone B at 20 µM bound covalently to both the AR AF-1 and GR AF-1 proteins that have limited similar amino acid residue sequence [19]. However, the interference of ligand-binding to all three steroid hormone receptors LBDs at 30 µM raised a concern about the reactivity of the enone group in niphatenone B. Alkylation experiments revealed that indeed both enantiomers of niphatenone B were reactive with glutathione while the unrelated covalent binder EPI-002 was not. Alkylation with glutathione suggests that niphatenones are reactive, lack specificity and are not a feasible scaffold for further drug development unless medicinal chemistry approaches can produce an analogue that is not a general alkylating agent yet still has specific activity to the AR NTD. Non-selective alkylators are quickly dismissed as potential drug candidates due to potential haptenization and toxicity. Here we show that niphatenones are reactive using the gold standard assay for alkylation, glutathione adduct formation.

## Supporting Information

Figure S1 **Effect of niphatenone B on Cos-1 proliferation.** Cos-1 cells treated under serum-free and phenol-red free conditions with 1.0 µM (S)- and (R)-niphatenone B or DMSO vehicle control for 24 h. Cells were labeled and BrdU incorporation was measured by ELISA. Data is presented as percentage of vehicle control. Bars represent the mean ± SEM of three independent experiments.
(EPS)

Figure S2 **LNCaP cells require forced expression of PR-β or GR to measure transcriptional activity in response to cognate ligands.** LNCaP cells co-transfected with empty vector or expression vector for PR-β. (A) or GR (B) and the corresponding luciferase reporter (PRE-Luc or GRE-Luc), under serum-free and phenol-red free conditions, were exposed to 10 nM of progesterone, dexamethasone or ethanol vehicle control for 48 h. Representative figures of three independent experiments. Bars represent the mean ± SD of three technical replicates.
(EPS)

Figure S3 **AR NTD inhibitor inhibits all AR species.** Androgen receptor (AR) transcriptional activity can be blocked by targeting either the LBD or the NTD. The first approach is the basis for development of abiraterone acetate and anti-androgens (AA) used in the clinic to treat prostate cancer. Abiraterone acetate blocks the synthesis of androgen. Both abiraterone acetate and antiandrogens that target the LBD have transient therapeutic effects. Eventually the cancer will become resistant to inhibitors of AR LBD possibly via expression of AR splice variants. On the other hand, by targeting the NTD which possesses most if not all the transcriptional activity of AR, an NTD inhibitor (NTDI) will effectively inhibit transcriptional activity of full-length AR, regardless of ligand binding, and truncated constitutively active AR splice variant lacking LBD.
(EPS)
